# *In situ* Immobilization of Copper Nanoparticles on Polydopamine Coated Graphene Oxide for H_2_O_2_ Determination

**DOI:** 10.1371/journal.pone.0157926

**Published:** 2016-07-05

**Authors:** Yingzhu Liu, Yanwei Han, Rongsheng Chen, Haijun Zhang, Simin Liu, Feng Liang

**Affiliations:** 1 The State Key Laboratory for Refractories and Metallurgy, School of Chemical Engineering and Technology, Wuhan University of Science and Technology, Wuhan 430081, China; 2 Key Laboratory of Analytical Chemistry for Biology and Medicine of Ministry of Education, Wuhan University, Wuhan 430072, China; 3 Key Laboratory of Inorganic Coating Materials, Chinese Academy of Sciences, Shanghai 200050, China; US Naval Reseach Laboratory, UNITED STATES

## Abstract

Nanostructured electrochemical sensors often suffer from irreversible aggregation and poor adhesion to the supporting materials, resulting in reduced sensitivity and selectivity over time. We describe a versatile method for fabrication of a H_2_O_2_ sensor by immobilizing copper nanoparticles (Cu NPs; 20 nm) on graphene oxide (GO) sheets *via in-situ* reduction of copper(II) on a polydopamine (PDA) coating on a glassy carbon electrode. The PDA film with its amino groups and catechol groups acts as both a reductant and an adhesive that warrants tight bonding between the Cu NPs and the support. The modified electrode, best operated at a working voltage of −0.4 V (vs. Ag/AgCl), has a linear response to H_2_O_2_ in the 5 μM to 12 mM concentration range, a sensitivity of 141.54 μA∙mM‾^1^∙cm‾^2^, a response time of 4 s, and a 1.4 μM detection limit (at an S/N ratio of 3). The sensor is highly reproducible and selective (with minimal interference to ascorbic acid and uric acid). The method was applied to the determination of H_2_O_2_ in sterilant by the standard addition method and gave recoveries between 97% and 99%.

## Introduction

Rapid, low-cost and reliable detection of hydrogen peroxide (H_2_O_2_) is of particular significance due to its wide application in food industry, environmental protection, and medical field [1–4]. Most previous studies indicate that H_2_O_2_ is actually a universal metabolic intermediate in organism and is closely associated with the metabolic function of human body [5, 6]. Moreover, bioaccumulation of H_2_O_2_ produces oxidative stress and results in severely detrimental damage to cells [7]. Nowadays many analytical methods have been employed for the determination of hydrogen peroxide, such as chemiluminescence, spectrophotometry, titrimetry and electrochemistry [8–11]. Among them, electrochemical sensors are attracted much attention due to its excellent properties of low-cost, simplicity, high sensitivity and handing convenience [12].

Hydrogen peroxide sensors based on the nanoparticles of metal or metal oxides have been intensively explored recently [13–15]. The electrocatalytic activities of the nanoparticles can produce remarkable voltammetric responses towards H_2_O_2_. But the irreversible aggregation will occur due to the high surface energy of free nanoparticles and will significantly decrease the analytical performance [16, 17]. A variety of supporting materials have been employed to disperse the nanoparticles. And graphene is the most frequently employed substrate to integrate the nanoparticles by procedures such as hydrothermal deposition and chemical vapor deposition [18, 19]. The sensitivity and selectivity of H_2_O_2_ determination depend on the immobilized nanoparticles. Therefore, the adhesion of the immobilized nanoparticles to the substrate is a key issue for H_2_O_2_ sensors in practical applications.

Herein, we present a versatile approach to immobilize nanoparticles on graphene sheets *via in situ* reduction of metal ions on the polydopamine (PDA) coating that can provide a tight bonding between the nanoparticles and the supporting materials. Graphene oxide (GO) was coated with a PDA film through simple dip-coating in an aqueous solution of dopamine, which can generate a nanofilm on most inorganic and organic materials in alkaline media by self-polymerization [20–23]. Dopamine is a chemical agent that contains catechol and amine groups. The PDA film composed of cross-linking with amine and catechol groups can serve as a reductant as well as an adhesive agent [24–28]. Copper nanoparticles (Cu NPs) were produced on the PDA film by *in situ* chemical reduction of copper ions and were integrated to the graphene sheets by the adhesive PDA coating. The GO/PDA/Cu NPs composite was characterized by transmission electron microscope (TEM) and X-ray photoelectron spectroscopy (XPS). The electrochemical behaviors were studied by cyclic voltammetry (CV) and amperometric i-t curve. The electrochemical sensor fabricated by GO/PDA/Cu NPs exhibited high sensitivity towards H_2_O_2_ determination with excellent reproducibility. The sensor also displayed good selectivity with minimal interference from the coexisting species such as ascorbic acid (AA) and uric acid (UA) in biological fluids and can be applied to real sample analysis.

## Materials and Methods

### Reagents

Hydrogen peroxide (H_2_O_2_, 30%) was purchased from Nangjing Chemical Reagent. Dopamine hydrochloride, tris(hydroxymethyl)aminomethane (Tris, 99%), ascorbic acid (AA), uric acid (UA) and polyvinylpyrrolidone (PVP, MW = 5000) were purchased from Sigma-Aldrich and used without further purification. Graphene oxide (GO, 1 mg/mL) was obtained from Institute of Coal Chemistry of Chinese Academy of Science. Other analytical grade chemicals were purchased from Sinopharm Group Co. Ltd. and also used without further purification. All aqueous solutions were prepared with deionized (DI) water and prepared just prior to use.

### Instruments

The microstructure and composition of GO/PDA and GO/PDA/Cu NPs were characterized by TEM (JEM-2010 UHR, JEOL) and XPS (ESCALAB 250, Thermo Fisher Scientific) using the Al Kα radiation (1486.6 eV, 15 kV, 150 W) at a vacuum of 2×10^−9^ mbar. Electrochemical measurement was performed at room temperature in phosphate buffer (PBS) on the CHI 660e potentiostat (CH Instruments Inc., Shanghai, China). A simple three-electrode configuration used in the measurement consisted of an Ag/AgCl electrode as the reference electrode, a Pt wire as the counter electrode, and a glassy carbon electrode (GCE, 3 mm in diameter) modified with GO/PDA/Cu NPs composite as the working electrode.

### Preparation of GO/PDA and GO/PDA/Cu NPs

**Fig 1:**
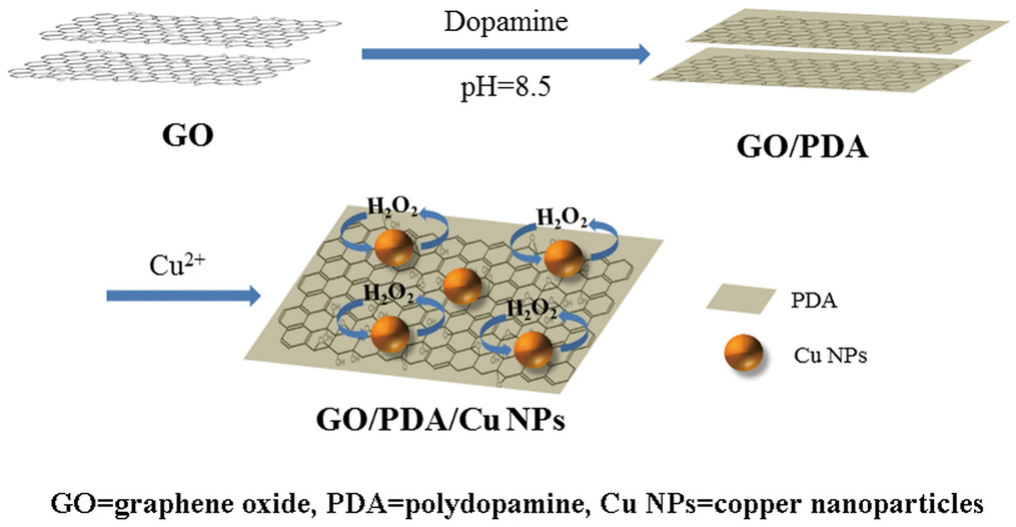
Scheme of the GO/PDA/Cu NPs sensor fabricated by immobilizing Cu NPs on GO sheets *via in-situ* reduction of copper(II) on a PDA coating.

GO powder was obtained by drying the purchased GO aqueous solution (1 mg/mL) at 80°C under vacuum. An amount of 20 mg GO powder was dispersed in 4 mL Tris buffer (10 mM, pH = 8.5) and ultra-sonicated in ice bath for 5 min. Then added 16 mL Tris buffer in the GO solution and continued ultra-sonicated for 5min to obtain a homogeneous dispersion of GO. The GO solution was separated by centrifugation before washing with Tris buffer for three times, and then dispersed in 2 mg/mL dopamine solution under stirring for 24 h at room temperature. GO/PDA composite was obtained by centrifugation and dried in vacuum at 60°C. An amount of 10 mg of GO/PDA composite was added into the fresh prepared 10 mM copper sulfate solution, followed by addition of sodium citrate (0.1 M) and PVP (1 wt.%) under stirring for 12 h. After centrifugation, the GO/PDA/Cu NPs sediment was washed with DI water and dried in vacuum [29]. The fabrication procedures can be depicted in Fig 1.

The GCE was polished with 0.05 μm α-Al_2_O_3_ power, and then sonicated in ethanol and DI water for several times to obtain a clean surface. The GO/PDA/Cu NPs composite was dripped onto the cleaned GCE surface. For comparison, GO/PDA composite was also dripped onto the GCE surface. The electrodes were dried at room temperature prior to electrochemical test.

## Results and Discussion

### Morphology and composition of GO/PDA/Cu NPs composite

**Fig 2:**
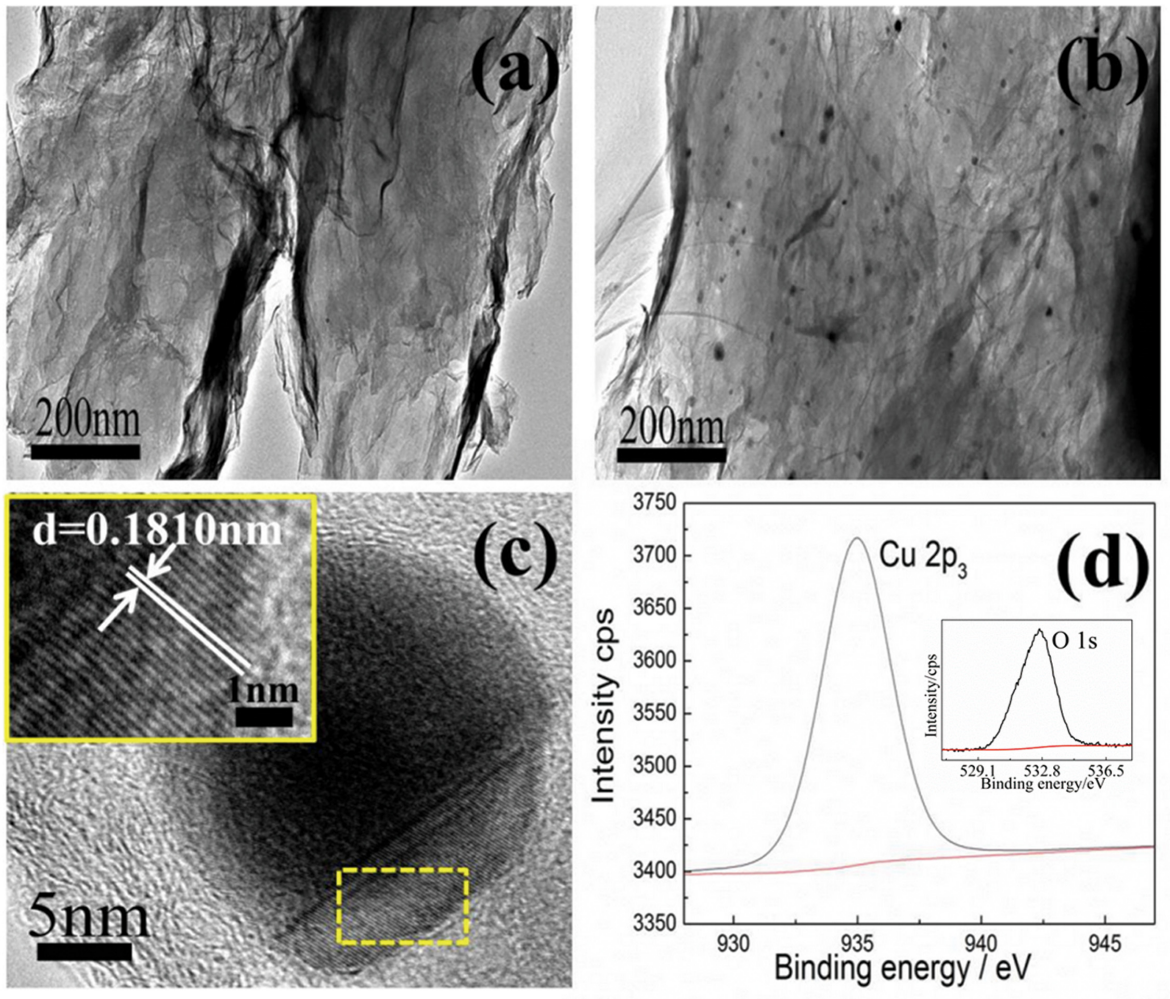
TEM images of GO/PDA (a), GO/PDA/Cu NPs (b), HR-TEM images of GO/PDA/Cu NPs (c) and the Cu2p and O 1s (inset) XPS spectra of GO/PDA/Cu NPs (d).

The morphology of GO/PDA and GO/PDA/Cu NPs was investigated by TEM. The TEM images revealed multilayer structures of GO/PDA composite without any particles (Fig 2a). Spherical nanoparticles with an average diameter of 20±4 nm (estimation from 100 nanoparticles) uniformly deposited on the surface of GO/PDA were observed in Fig 2b. As can be seen from HR-TEM images (inset in Fig 2c), the spacing of the lattice fringes (0.1810 nm) corresponds to the lattice planes Cu (1, 1, 1) [30], suggesting the deposition of Cu NPs on the GO/PDA sheets. Chemical composition of the nanoparticles was also analyzed by XPS (Fig 2d). The Cu 2p_3_ single peak for Cu NPs at 934.92 eV should be attributed to copper zero or copper (I). The O 1s peak at 532.67 eV for Cu NPs indicates the existence of copper oxide [31]. The results further confirm that the formation of the GO/PDA/Cu NPs composite.

### Electrochemical behaviors of the GO/PDA/Cu NP sensor

**Fig 3:**
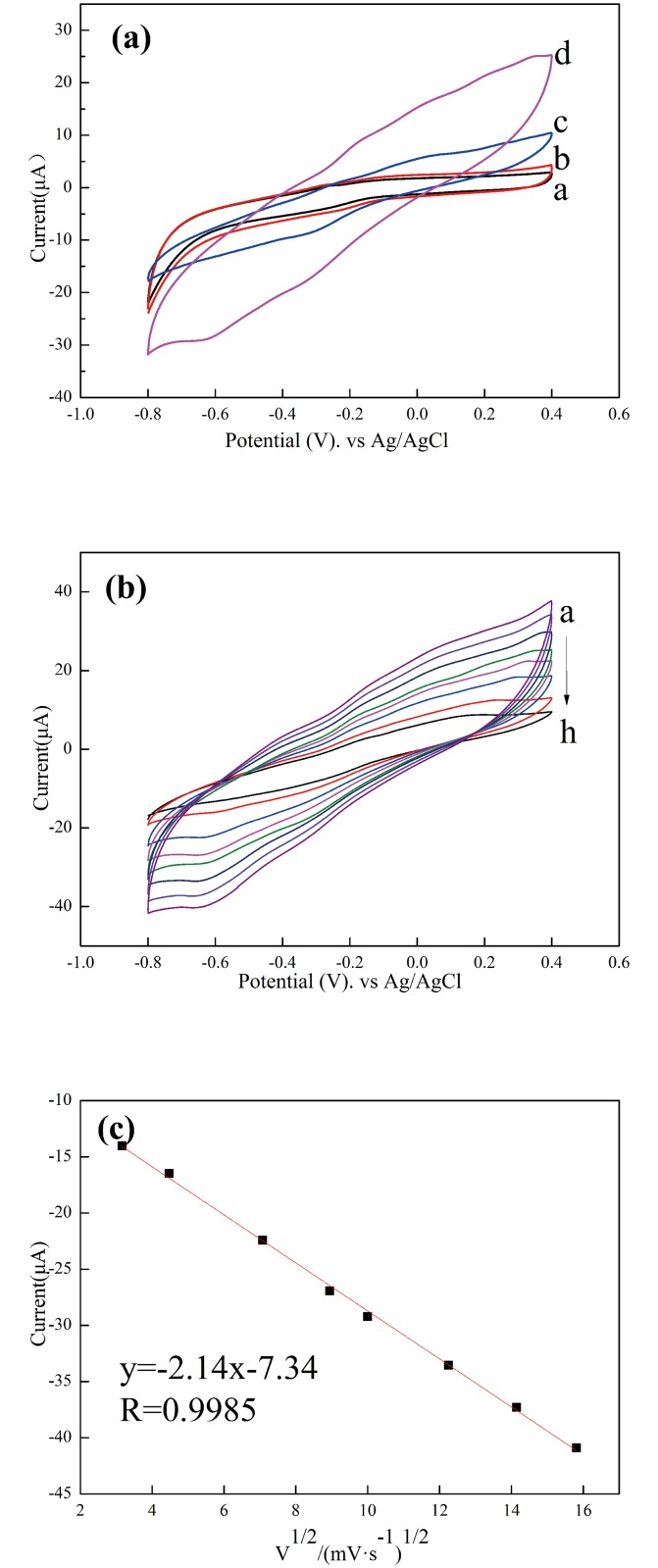
(a) Cyclic voltammograms of GO/PDA sensor (a and b) and GO/PDA/Cu NPs/GCE sensor (c and d) in the absence (a and c) and presence (b and d) of 10 mM H_2_O_2_ at a scanning rate of 100 mV/s in 0.1 M pH 7.2 PBS. (b) Cyclic voltammograms of the GO/PDA/Cu NP sensor in 0.1 M pH 7.2 PBS containing 10 mM H_2_O_2_ at different scan rates (V) of 10, 20, 50, 80, 100, 150, 200 and 250 mV/s (from a to h). (c) Plots of the response peak current vs. the square root of the scan rate (V^1/2^). Results are presented as mean ±SD (error bar) of triplicate experiments.

Cyclic voltammograms (CV) were measured to investigate the electrocatalytic property of the GO/PDA/Cu NPs composite in PBS buffer, as shown in Fig 3a. No obvious current response changes can be observed between the CV of GO/PDA in the absence (a) and presence (b) of 10 mM H_2_O_2_. Compared the GO/PDA/Cu NPs without H_2_O_2_ (c), the GO/PDA/Cu NPs with H_2_O_2_ (d) shows a steep increase of current response. There are no obvious current peaks associated with CuNPs or PDA, as can be seen from Fig 3a in the absence of H_2_O_2_. While in the presence of H_2_O_2_, a peak current at -0.65 V associated with the reduction of H_2_O_2_ was observed at the GO/PDA/Cu NPs sensor. The odd shape of the CVs should be attributed to the impedence of the PDA film that produced an ohmic shape when the current was significantly enhanced in the presence of H_2_O_2_ at the GO/PDA/Cu NPs sensor [32–33]. These results indicate that the GO/PDA/Cu NPs composite exhibits good electrocatalytic performance for the reduction of H_2_O_2_. Thus it can be considered as for a promising H_2_O_2_ sensor. The electrocatalytic detection of H_2_O_2_ should be attributed to the reduction of H_2_O_2_ on the GO/PDA/Cu NPs surface. The possible reaction can be summarized as the following equation [14, 34]:

$${\text{H}}_{\text{2}}{\text{O}}_{\text{2}}\to {\text{H}}_{\text{2}}\text{O}+1/2{\text{ O}}_{\text{2}}$$

Fig 3b shows the cyclic voltammograms of GO/PDA/Cu NPs sensor in 0.1 M pH 7.2 PBS containing 10 mM H_2_O_2_ at different scan rates of 10~250 mV/s. As can be seen, the response currents are proportional to the square root of the scan rate with the correlation coefficient of 0.9985 (Fig 3c), indicating that the electrocatalytic reaction on the modified electrode is a diffusion-controlled process instead of a surface-controlled one [35].

### Determination of H_2_O_2_ by the GO/PDA/Cu NP sensor

**Fig 4:**
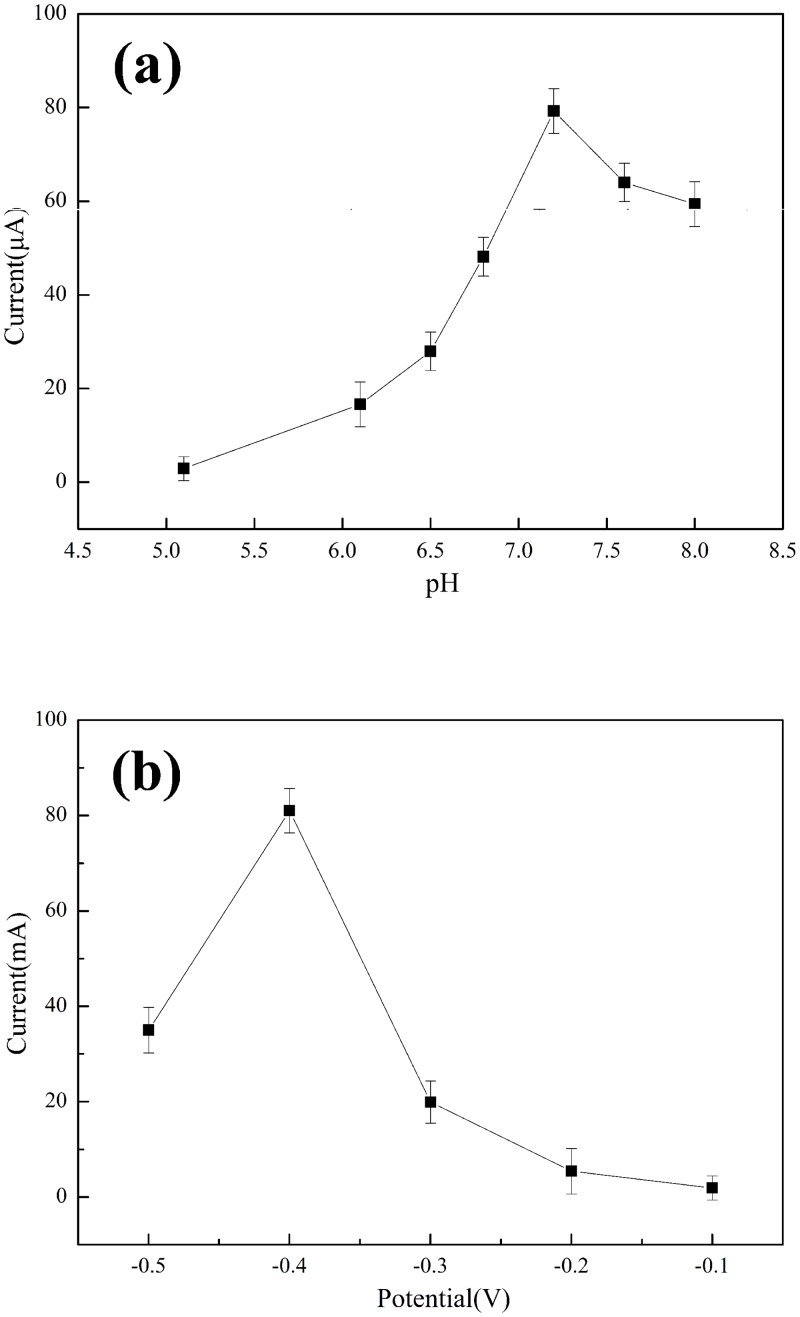
Effects of pH value at an applied potential −0.4 V (vs. Ag/AgCl) (a) and applied potential (b) on the response current of 10 mM H_2_O_2_ on the GO/PDA/Cu NP sensor. Results are presented as mean ±SD (error bar) of triplicate experiments.

The pH value and applied potential are essential parameters to electrocatalytic process of GO/PDA/Cu NP sensor. Hence, effects of pH (Fig 4a) and applied potential (Fig 4b) on the response current of 10 mM H_2_O_2_ at GO/PDA/Cu NP sensor were studied. In Fig 4a, the current response increases when the pH increases from 5.1 to 7.2, and then begins to decrease when the pH is further increasing. It indicates that pH 7.2 is optimal condition for the electrocatalytic reaction, which is close to the pH in the environment of human body fluids. In Fig 4b, it is found that the current response increases with the increase of applied potential in the range of −0.1 V and −0.4 V. It suggests that the current response of the GO/PDA/Cu NP sensor has reached high electrocatalytic activity at the applied potential of −0.4 V. For a higher sensitivity, −0.4 V was chosen as the optimal applied potential for the detection of hydrogen peroxide.

**Fig 5:**
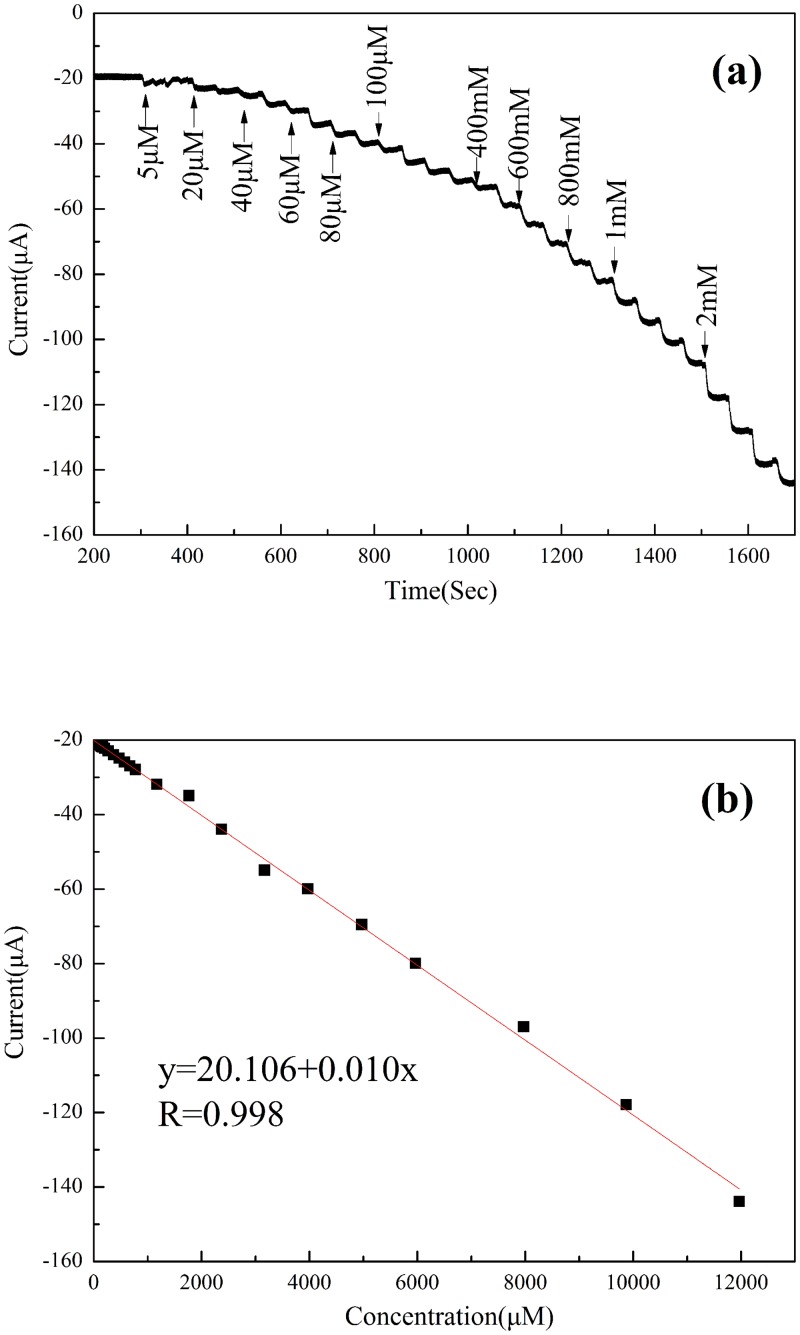
(a) Amperometric responses of the GO/PDA/Cu NP sensor upon successive addition of H_2_O_2_ into 0.1 M pH 7.2 PBS at an applied potential −0.4 V under a stirring condition. (b) Calibration curve of the GO/PDA/Cu NP sensor versus H_2_O_2_ concentration. Results are presented as mean ±SD (error bar) of triplicate experiments.

The electrocatalytic responses of the GO/PDA/Cu NP sensor to hydrogen peroxide were further evaluated by amperometric current-time response upon successive addition of H_2_O_2_ into 0.1 M pH 7.2 PBS with an applied potential −0.4 V under a stirring condition (Fig 5a). By increasing the concentration of H_2_O_2_, the current response increases gradually as shown in Fig 5b. A linear relationship between hydrogen peroxide concentration (x) and current response (y) is obtained as a function of hydrogen peroxide in the concentration range of 5 μM to 12 mM with the calibration equation of y = 20.106+0.010x (R = 0.998). The estimated sensitivity for the hydrogen peroxide sensor is 141.54 μA·cm^−2^·mM^−1^, and the detection limit is calculated to be 1.4 μM at an S/N = 3 with a response time of 4 s.

### Reproducibility, stability and selectivity

**Fig 6:**
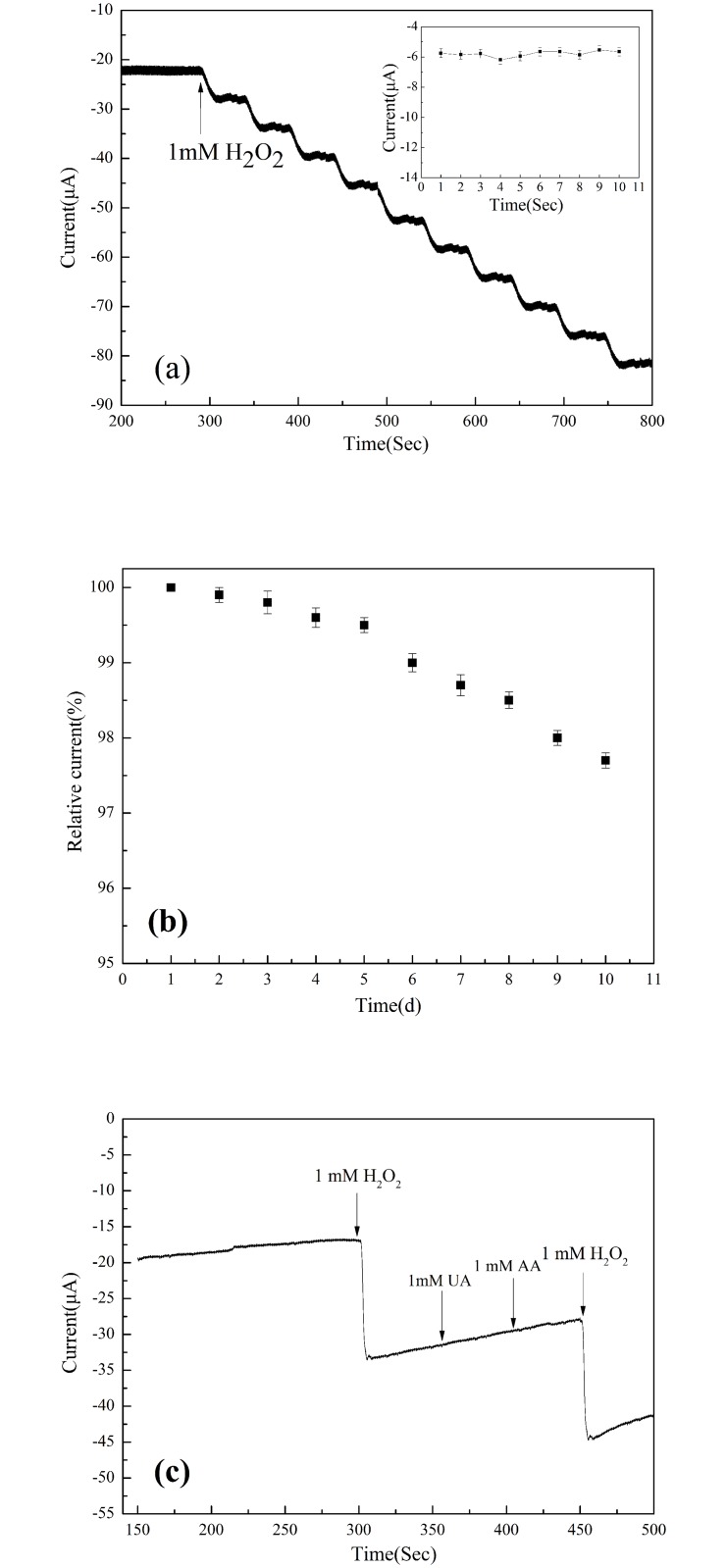
(a) Amperometric responses for successive injection of 1 mM H_2_O_2_ into 0.1 M pH 7.2 PBS with continuous stirring at an applied potential of −0.4 V with the inset showing the amperometric response for different injections. (b) Long-term stability of the sensor in 10 days. (c) Amperometric responses of the GO/PDA/Cu NP sensor upon the successive addition of 1 mM H_2_O_2_, UA, AA and H_2_O_2_ into 0.1 M pH 7.2 PBS at an applied potential −0.4 V under a stirring condition. Results are presented as mean ±SD (error bar) of triplicate experiments.

In order to investigate the reproducibility of the GO/PDA/Cu NP sensor, amperometric responses for successive injection of 1 mM H_2_O_2_ into 0.1 M pH 7.2 PBS with continuous stirring at an applied potential of −0.4 V were examined and the results are shown in Fig 6a. The inset of Fig 6a shows the amperometric response for different injections. The relative standard deviation (RSD) is calculated to be 2.5%,indicating good reproducibility of the GO/PDA/Cu NP sensor. Also, the stability of the GO/PDA/Cu NP sensor was evaluated by successive monitoring after storing in desiccator for 10 days, as seen in Fig 6b. It can be seen that only 2.3% of the current signal diminished from the original current response, indicating that the sensor is considerably stable. Thus, the GO/PDA/Cu NP sensor has good reproducibility and stability towards hydrogen peroxide determination.

To evaluate the selectivity of the GO/PDA/Cu NP sensor, UA and AA were employed as interfering species by successive addition of 1 mM H_2_O_2_, UA, AA and H_2_O_2_ into 0.1 M pH 7.2 phosphate buffer with an applied potential −0.4 V under a stirring condition (Fig 6c). Compared to the current response of H_2_O_2_, the current responses of UA and AA were negligible, revealing that these substances don’t interfere the detection of hydrogen peroxide.

### Real sample analysis

**Fig 7:**
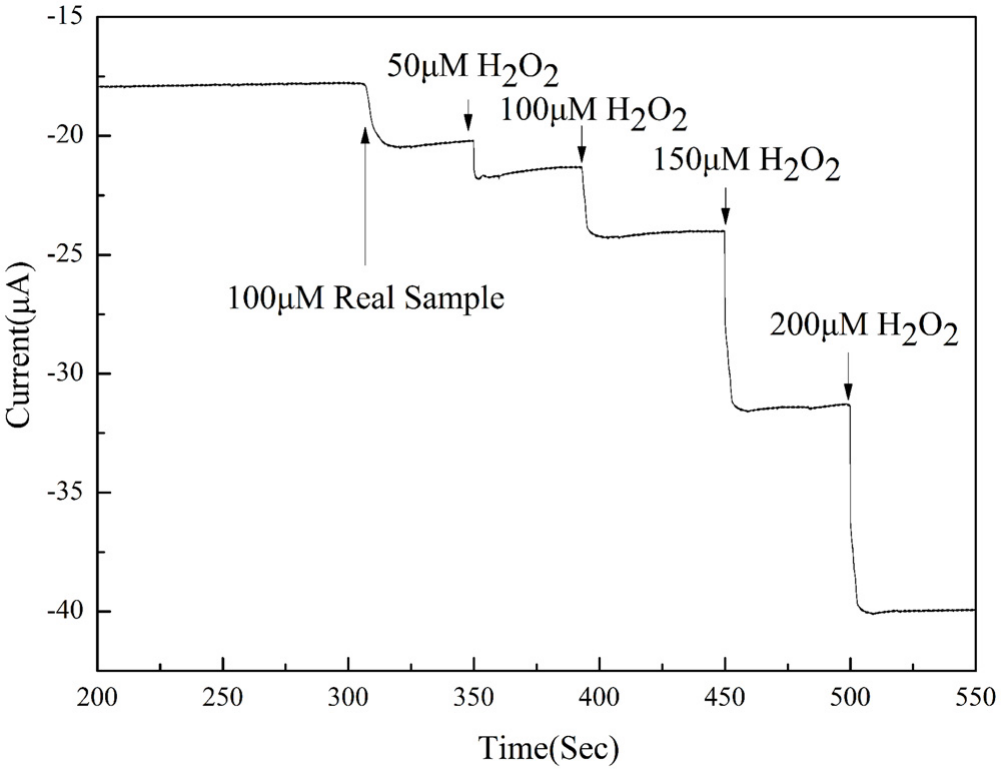
Amperometric responses of the GO/PDA/Cu NP sensor upon successive addition of 100 μM real sample, 50 μM H_2_O_2_, 100 μM H_2_O_2_, 150 μM H_2_O_2_ and 200 μM H_2_O_2_ into 0.1 M pH 7.2 PBS with an applied potential −0.4 V under a stirring condition.

To test the reliability of the GO/PDA/Cu NP sensor as a non-enzymatic electrochemical hydrogen peroxide sensor, amperometric measurement of H_2_O_2_ in sterilant obtained from a local supermarket was carried out. Before measurement, the sterilant sample was diluted 10000-fold with phosphate buffer. In Fig 7, amperometric responses of the GO/PDA/Cu NP sensor upon the successive addition of 100 μM real sample, 50 μM H_2_O_2_, 100 μM H_2_O_2_, 150 μM H_2_O_2_ and 200 μM H_2_O_2_ into 0.1 M pH 7.2 PBS with an applied potential −0.4 V under a stirring condition are displayed. Then contact lens solution with 1000-fold dilution and milk sample with 50-fold dilution are also examined. The concentration of H_2_O_2_ was calculated by standard addition method [36] and they are summarized in Table 1. The recovery values for the samples were 95%-99%. According to the obtained results, the GO/PDA/Cu NP sensor exhibits excellent electrocatalytic performance in practical analysis.

**Table 1:** Amperometric determination of H_2_O_2_ in sterilant (1), contact lens solution (2), and the milk sample (3).

Sample	Value found in real sample (μM)	Added H_2_O_2_ (μM)	Found H_2_O_2_ (μM)	Recovery (%)
1	100	-	-	-
-	-	50	49	98
-	-	100	99	99
-	-	150	146	97
-	-	200	198	99
2	150	-	-	-
-	-	50	49.5	99
-	-	100	97	97
-	-	150	147	98
-	-	200	192	96
3	10	-	-	-
-	-	20	19.6	98
-	-	50	48	96
-	-	70	66.5	95
-	-	100	97	97

## Conclusions

We present a versatile approach to fabricate H_2_O_2_ sensor by immobilization of Cu NPs on the sheets of GO *via in situ* reduction of copper ions on the PDA coating that can provide a tight bonding between the Cu NPs and GO. In this process, the PDA coating acts as a reductant as well as an adhesive agent. The GO/PDA/Cu NPs composite exhibits high sensitivity towards H_2_O_2_ determination with excellent reproducibility. The sensor also shows good selectivity with minimal interference from the coexisting species such as AA and UA in biological fluids. The recovery values estimated from the standard addition method imply the potential applications of the GO/PDA/Cu NP sensor in practical analysis.
